# A phase I trial evaluating the safety and immunogenicity of a candidate tuberculosis vaccination regimen, ChAdOx1 85A prime – MVA85A boost in healthy UK adults

**DOI:** 10.1016/j.vaccine.2019.10.102

**Published:** 2020-01-22

**Authors:** Morven Wilkie, Iman Satti, Alice Minhinnick, Stephanie Harris, Michael Riste, Raquel Lopez Ramon, Sharon Sheehan, Zita-Rose Manjaly Thomas, Daniel Wright, Lisa Stockdale, Ali Hamidi, Matthew K. O'Shea, Kritica Dwivedi, Hannah Michaela Behrens, Tamara Davenne, Joshua Morton, Samantha Vermaak, Alison Lawrie, Paul Moss, Helen McShane

**Affiliations:** a The Jenner Institute, University of Oxford, Oxford OX3 7DQ, UK; b Institute of Immunology and Immunotherapy, University of Birmingham, Edgbaston, Birmingham B15 2TT, UK

**Keywords:** Tuberculosis, Vaccine, ChAdOx1 85A, MVA85A, Safety, Immunogenicity

## Abstract

**Background:**

This phase I trial evaluated the safety and immunogenicity of a candidate tuberculosis vaccination regimen, ChAdOx1 85A prime-MVA85A boost, previously demonstrated to be protective in animal studies, in healthy UK adults.

**Methods:**

We enrolled 42 healthy, BCG-vaccinated adults into 4 groups: low dose Starter Group (n = 6; ChAdOx1 85A alone), high dose groups; Group A (n = 12; ChAdOx1 85A), Group B (n = 12; ChAdOx1 85A prime – MVA85A boost) or Group C (n = 12; ChAdOx1 85A – ChAdOx1 85A prime – MVA85A boost). Safety was determined by collection of solicited and unsolicited vaccine-related adverse events (AEs). Immunogenicity was measured by antigen-specific *ex-vivo* IFN-γ ELISpot, IgG serum ELISA, and antigen-specific intracellular IFN-γ, TNF-α, IL-2 and IL-17.

**Results:**

AEs were mostly mild/moderate, with no Serious Adverse Events. ChAdOx1 85A induced Ag85A-specific ELISpot and intracellular cytokine CD4+ and CD8+ T cell responses, which were not boosted by a second dose, but were boosted with MVA85A. Polyfunctional CD4+ T cells (IFN-γ, TNF-α and IL-2) and IFN-γ+, TNF-α+ CD8+ T cells were induced by ChAdOx1 85A and boosted by MVA85A. ChAdOx1 85A induced serum Ag85A IgG responses which were boosted by MVA85A.

**Conclusion:**

A ChAdOx1 85A prime – MVA85A boost is well tolerated and immunogenic in healthy UK adults.

## 1 Background

Tuberculosis (TB) is now the leading single infectious disease killer in the world despite an annual 2% reduction in TB worldwide. In 2017, an estimated 1.6 million people died from TB, with 10 million new cases throughout the world [1]. The End TB strategy of global eradication of TB seeks to address this pressing issue, and one of the most valuable tools for its success is the development of a safe and effective TB vaccine [1].

The only licenced TB vaccine, Bacille Calmette-Guérin (BCG), is highly effective at preventing TB meningitis and disseminated disease in children. However, the lack of consistent protection against adult pulmonary TB means a more universally effective vaccine is urgently needed. Subunit vaccines to boost the efficacy of BCG allow the retention of the protective benefits against systemic disease [2], [3], [4], [5]. Recombinant viral vectors have shown early promise in animal and human studies [6], [7], [8], [9], [10], however the first efficacy trial with recombinant Modified Vaccinia virus Ankara (MVA85A) expressing the highly conserved, immunodominant, *Mycobacterial* antigen 85A (Ag85A) did not confer additional protection over BCG alone in South African infants [11]. This was likely in part due to reduced immunogenicity seen in this setting. More potent regimens are therefore needed. Heterologous ‘prime-boost’ vaccination strategies, using different viral vectored vaccines expressing proteins from malaria, have shown strong and sustained cellular immune responses correlating with malaria protection [12], [13]. A recombinant adenoviral vaccine candidate, AERAS-402, has been evaluated as a prime-boost regimen with MVA85A in a phase I clinical trial in healthy BCG-vaccinated UK adults. This heterologous prime-boost regime was well-tolerated and induced increased frequency of antigen-specific CD4+ and CD8+ T-cell responses [14]. Recently, a protein adjuvanted subunit vaccine M72, conferred 54% protection against TB disease in *M.tb* latently infected subjects [15].

ChAdOx1 85A is a novel chimpanzee adenoviral vectored vaccine expressing Ag85A. An advantage of simian adenoviral vectors over human adenoviral vectors is the low prevalence of pre-existing anti-vector antibodies in humans, a factor limiting the use of adenoviruses to date [16]. The vector, ChAdOx1, expressing two influenza antigens, NP and M1, has previously been evaluated in a small phase I dose escalation trial and was found to be safe and immunogenic [17]. Preclinical murine studies have demonstrated ChAdOx1 85A to be consistently protective when used as part of a BCG-ChAdOx1 85A-MVA85A immunisation regime [18]. The aim of this first-in-human experimental medicine trial was to evaluate the safety and immunogenicity of ChAdOx1 85A, alone and as a prime-boost regime with MVA85A in healthy, BCG-vaccinated UK adults.

## 2 Methods

### 2.1 Trial design

We conducted a phase I, open label, dose escalation clinical trial in 42 BCG-vaccinated UK adults. All trial documents were approved by the Medicines and Healthcare Regulatory Agency (MHRA, EudraCT 2012-005118-21) and South Central – Oxford A Research Ethics Committee (reference: 13/SC/0098). It was registered with clinicaltrials.gov (ref NCT01829490) and conducted according to the principles of the Declaration of Helsinki and Good Clinical Practice.

### 2.2 Participants

Volunteers were recruited from the Oxford and Birmingham area, providing written informed consent prior to screening. Visits occurred at the Centre for Clinical Vaccinology and Tropical Medicine (CCVTM), Oxford, and the NIHR Wellcome Trust Clinical Research Facility, Birmingham (NIHR WTCRF). All vaccination visits were at the CCVTM. Volunteers were healthy, aged between 18 and 55, and had received BCG vaccination from an independent source prior to screening (no less than 6 months prior to date of enrolment). Baseline biochemical and haematological analysis, serological testing for HIV, hepatitis B and hepatitis C were performed to ensure no abnormalities warranting exclusion. Latent *Mycobacterium tuberculosis* (*M.tb*) infection (LTBI) was excluded at screening with a negative IFN-γ release assay response to ESAT-6 and CFP-10, by means of the T Spot.TB kit (Oxford Immunotec, UK) or QuantiFERON®-TB Gold In-Tube. The full inclusion and exclusion criteria are in Supplementary Methods 1.

### 2.3 Vaccines

ChAdOx1 85A (lot no. 02N12-01) was constructed under Good Manufacturing Practice (GMP) conditions at the Clinical Biomanufacturing Facility (CBF), University of Oxford, UK [16]. MVA85A (lot no. 0050811) was constructed as previously documented [18], and produced under GMP conditions by IDT Biologika GmbH (IDT), Germany. Both vaccines were stored prior to use at the CCVTM in a secure locked, monitored −80 °C freezer. Both vaccines were administered intramuscularly in the deltoid region of the arm.

### 2.4 Clinical interventions

Six volunteers were enrolled into the Starter Group and received ChAdOx1 85A at a dose of 5 × 10^9^ virus particles (vp) on day 0 (D0), a safety review was performed 48 h following vaccination of the first volunteer before vaccination of the subsequent five volunteers. A safety review was performed once all volunteers in this group had reached D14 to ensure no safety concerns before proceeding to the predicted optimal dose of ChAdOx1 85A based on the previous Flu study [17]. After successful safety review, the first Group A volunteer received ChAdOx1 85A at a dose of 2.5 × 10^10^ vp with safety review 48 h later. No safety concerns were raised, and a further 23 volunteers were randomised to Group A (ChAdOx1 85A 2.5 × 10^10^ vp at D0) or Group B (ChAdOx1 85A 2.5 × 10^10^ vp at D0 with MVA85A 1 × 10^8^ plaque forming units (pfu) boost at D56) using sequentially numbered, sealed envelopes prepared by an independent statistician and opened by the trial clinician at the time of volunteer enrolment. Following our previous trial results, where two doses of AERAS-402 followed by MVA85A was the most potent regime for the induction of antigen-specific CD8+ T cell responses [14], we added Group C, whereby 12 volunteers were enrolled to receive two doses of ChAdOx1 85A 2.5 × 10^10^ vp (D0 and D28) with MVA85A 1 × 10^8^ pfu boost at D119.

All volunteers had a clinical review 30 and 60 min following vaccination. Expected local and systemic solicited and unsolicited adverse events (AEs) were collected by volunteers using a 7-day diary-card, and reviewed by the clinical team at follow-up visits along with vital signs. Starter Group and Group A were followed-up at: D2, D14, D28, D56, D84 and D168, Group B: D2, D14, D28, D56, D58, D63, D84, D140 and D224, and Group C: D2, D14, D28, D30, D42, D56, D119, D121, D126, D147, D203 and D287. Solicited and unsolicited AEs were graded mild, moderate or severe. Assignment of causality was conducted according to a pre-defined criteria specified in the protocol. Safety bloods were taken 14 days following ChAdOx1 85A and 7 days following MVA85A, with bloods also taken on the day of 2nd and 3rd vaccinations depending on the individual volunteer schedule. Blood for exploratory immunology was taken at all visits.

### 2.5 Enzyme-Linked ImmunoSpot (ELISpot)

IFN-γ *ex-vivo* ELISpot was performed on freshly isolated Peripheral Blood Mononuclear Cells (PBMC) from volunteers in the Starter Group and Group A at: D0, D14, D28, D56, D84 and D168, Group B at: D0, D14, D28, D56, D63, D84, D140 and D224 and Group C at: D0, D14, D28, D42, D56, D119, D126, D147, D203 and D287, as previously described [14] using the Human IFN-γ ELISpot (ALP) kit (Mabtech).

Ag85A-specific responses were measured using a single pool of Ag85A peptides (66 15mer peptides, overlapping by 10 amino acids). Anti-vector responses were measured using ChAdOx1 (2 IU:1 PBMC) (Vector Core Facility, The Jenner Institute, Oxford, UK) and responses to MVA were measured using separate pools of CD4 (27 14-21mer peptides) and CD8 (36 9mer peptides) epitopes from Vaccinia and MVA (Peptide Protein research, UK) (final concentration 2 μg/ml) and Purified Protein Derivative (PPD) (Statens Serum Institute, Denmark) (20 μg/ml). Staphylococcal enterotoxin B (SEB) (Sigma) (10 μg/ml) was used as a positive control and unstimulated PBMC as a negative control.

### 2.6 Whole blood intracellular cytokine staining (WB ICS)

WB cytokine responses were measured in samples collected at D0, D14 and D168 (Group A); D0, D14, D56, D63 and D224 (Group B); and D0, D14, D28, D42, D119, D126 and D203 (Group C). The assay was performed on fresh samples as previously described [19]. Briefly, 500 µl WB was added to duplicate tubes containing either a pool of Ag85A peptides (66 15mer peptides, overlapping by 10 amino acids) at a final concentration of 2ug/ml, SEB at a final concentration of 5ug/ml (positive control) or tubes containing no antigen (negative control). The co-stimulatory antibodies α-CD28 and α-CD49d were added to each tube at a final concentration of 0.5 µg/ml and tubes were then incubated at 37 °C, 5% CO_2_ for 5 h before Brefeldin A was added at a final concentration of 5ug/ml. Tubes were then incubated at 37 °C in a water bath timed to switch off after 6 h. The following morning, 50ul 20 mM EDTA was added to each tube and incubated at room temperature for 15 mins. Duplicate tubes were then added to 9 ml FACS lysing solution (BD Bioscience) and incubated at room temperature in the dark for 10 mins before centrifuging at 1500 rpm for 7 mins. Supernatant was discarded and cell pellet washed in 10 ml PBS and centrifuged at 1500 rpm for 7 mins. Supernatant was discarded and the cell pellet resuspended in 1 ml PBS with 10% DMSO and transferred to a cryovial for freezing and batched analysis at the end of the trial.

**Supplementary figure 1:**
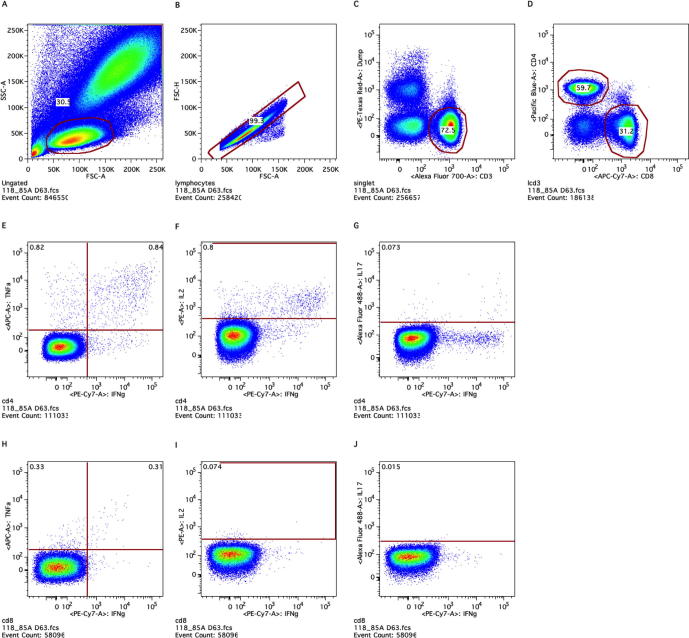
Gating strategy: Stimulated, fixed, lysed and stained whole blood samples were acquired on LSRII cytometer (BD) and data was analysed using Flowjo v8.8.7 (Treestar). Singlet, CD3+ and CD14-/CD19- lymphocytes were chosen (A-C). CD4+ and CD8+ T cells were gated on the CD3+ T cells (D). CD4+ and CD8+ T cells cytokines were gated (E-G and H-J respectively).

Samples were thawed, permeabilised and incubated with antibodies against CD3 (AF700), IFN-γ (PE–Cy7) (Ebioscience), CD4 (Pacific Blue), TNF-α (AF647), IL-17 (AF488) (Biolegend), CD8 (APC-H7) (Becton Dickinson), IL-2 (PE), CD14 (ECD) and CD19 (ECD) (Beckman Coulter). Data was acquired on an LSRII (Becton Dickinson), Instrument daily performance was monitored using Cytometer Setting and Tracking Beads. Data was analysed using FlowJo (Tree Star). CD4+ and CD8+ T cell cytokine responses were measured in singlet CD14-CD19-CD3+ cells (Supplementary Figure 1). Polyfunctional cytokine immune responses were analyzed using Pestle and Spice software packages (http://exon.niaid.nih.gov/spice/). Antigen-specific cytokine responses are presented by subtracting background unstimulated responses. Total summed cytokines (IFN-γ, TNF-α, IL-2 and IL-17 for CD4+ T cells and IFN-γ and TNF-α for CD8+ T cells) responses are presented.

### 2.7 Enzyme-linked immunosorbent assay (ELISA)

Immunoglobulin G (IgG) levels were measured in serum collected on D0, D14, D28, D56, D84 and D168 (Starter Group and Group A), at D0, D14, D28, D56, D63, D84, D140 and D224 (Group B) and at D0, D14, D28, D42, D56, D119, D126, D147 and D287 (Group C). Recombinant Ag85A (Lionex, Germany) was used to measure Ag85A-specific IgG responses. Non-recombinant MVA (1 × 10^7^pfu/ml) and ChAdOx1 (5 × 10^6^ IU/ml) (Vector Core Facility, Jenner Institute, Oxford) were used to measure vector-specific IgG responses.

The assay was performed in triplicate with serum diluted 1:10 in Casein blocking buffer (Sigma). Serum pooled from five high IgG responders was the positive control with serum from BCG-naïve individuals as negative control. Goat anti-human γ-chain whole IgG alkaline phosphatase conjugate (Sigma Aldrich) was used for capture of bound serum IgG. pNPP substrate (Sigma) was used for detection of IgG and the reaction stopped with 3 M NaOH. Absorbance was measured at 405 nm. Mean optical density (OD) of triplicate blank wells were subtracted from mean OD of test sample triplicate.

### 2.8 Statistical analysis

Safety was assessed by analysing frequency and severity of vaccine related local and systemic, solicited and unsolicited AEs, and summarised by frequency and severity of AEs using descriptive statistics. Baseline demographics, AEs, ELISpot, WB ICS and antibody data were analysed using GraphPad Prism. One way ANOVA was used to detect differences in baseline demographics across groups. The Mann Whitney *U* test was used to detect differences between groups, the Kruskal-Wallis test was used for looking at differences between more than two groups and the Wilcoxon matched-pairs signed rank test was used for differences between time points within the same group. Area Under the Curve (AUC) analysis was used to detect difference over time.

## 3 Results

**Fig. 1:**
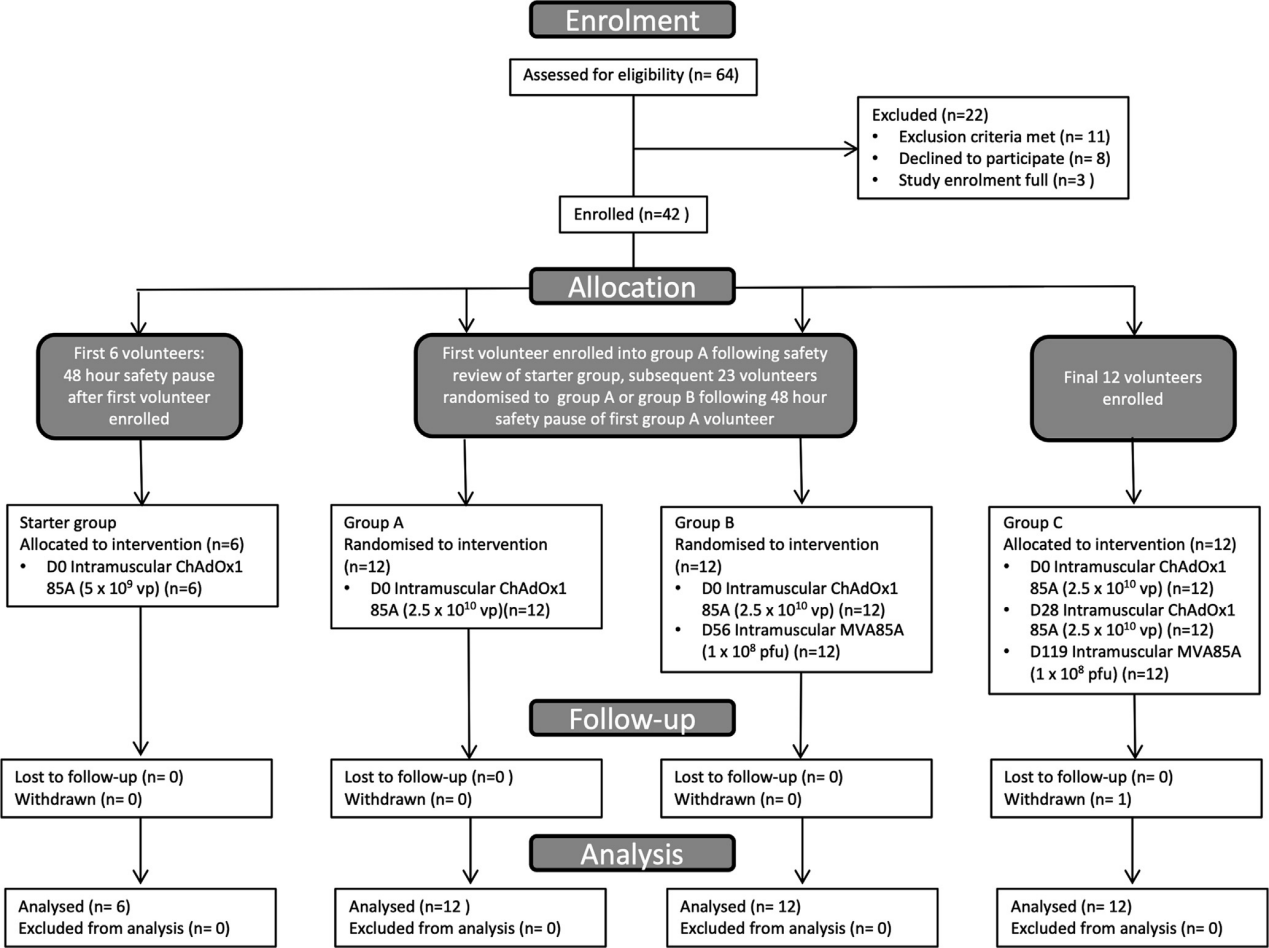
Study profile – CONSORT flow diagram showing volunteer recruitment and follow-up. Starter Group (5 × 10^9^ vp ChAdOx1 85A) enrolled first and following D14 safety review, first group A volunteer (2.5 × 10^10^ vp ChAdOx1 85A) enrolled. Following 48 h safety review of this volunteer, a further 23 volunteers subsequently randomised to group A or group B. Additional 12 volunteers enrolled into group C. One group C volunteer did not complete follow-up as withdrew from study prior to final visit; volunteer data included in analysis as all visits except D287 complete.

Between 22nd July 2013 and 30th June 2015, 42 volunteers were enrolled (Fig. 1 Consort diagram). One volunteer in Group C withdrew prior to their final visit; all data prior to the point of withdrawal were included in analysis. Baseline demographics including age, sex and time between BCG and enrolment, were comparable between groups (Supplementary Table 1).

### 3.1 Vaccine safety

**Supplementary figure 2:**
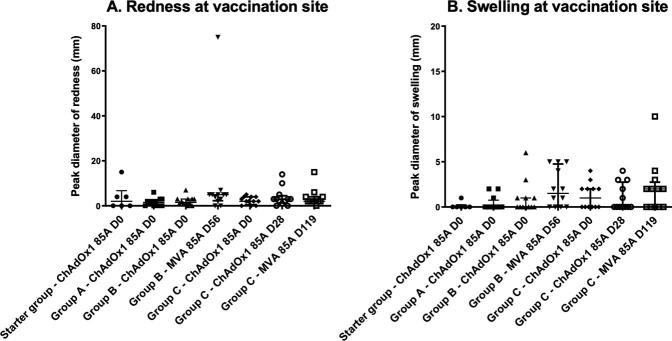
Local, solicited, vaccine reactogenicity. Figure shows peak diameter of redness (A) and swelling (B) at site of intramuscular vaccination in the 28 days following each vaccination with median and IQR shown.

There were no safety concerns in the Starter Group. Local and systemic AEs were expected and mild in nature (Table 1). Volunteers enrolled in Groups A, B and C reported expected local AEs, the majority of which were mild/moderate in nature (Table 1). Two volunteers in Group B reported severe pain at vaccination site following MVA85A vaccination at D56. Local redness and swelling across all vaccinations were comparable with no significant difference seen (p > 0.05) (Supplementary Fig. 2). The majority of systemic, solicited AEs were also mild to moderate in nature. Of those who reported a severe systemic AE (n = 4); one volunteer in Group A reported severe fatigue, feverishness and headache, and one in Group B reported severe feverishness and fatigue, all following D0 ChAdOx1 85A. One Group B volunteer reported severe feverishness, myalgia and malaise following MVA85A, and a second volunteer, also in Group B reported severe headache.

**Table 1:** Vaccine related AEs: Shown are the local and systemic solicited AEs which occurred following vaccination. Frequency is calculated as the number of volunteers counted once for each AE at maximal severity grading following each vaccination.

	Starter Group	Group A	Group B		Group C		
	*ChAdOx1 85A (D0)*	*ChAdOx1 85A (D0)*	*ChAdOx1 85A (D0)*	*MVA85A (D56)*	*ChAdOx1 85A (D0)*	*ChAdOx1 85A (D28)*	*MVA85A (D119)*
Local AE	n = 6	n = 12	n = 12	n = 12	n = 12	n = 12	n = 12
Pain							
*Mild*	4 (67)	7 (58)	8 (67)	2 (17)	10 (83)	6 (50)	8 (67)
*Moderate*		1 (8)	3 (25)	8 (67)		1 (8)	4 (33)
*Severe*				2 (17)			
Redness							
*Mild*	3 (50)	7 (58)	10 (83)	9 (75)	9 (75)	10 (83)	11 (92)
*Moderate*				1 (8)			
*Severe*							
Swelling							
*Mild*	1 (17)	3 (25)	5 (42)	8 (67)	6 (50)	5 (42)	7 (58)
*Moderate*							
*Severe*							
Scaling							
*Yes*				1 (8)			
Warmth							
*Mild*	1 (17)	4 (33)	2 (17)	3 (25)	5 (42)	1 (8)	4 (33)
*Moderate*							
*Severe*							
Itch							
*Mild*		3 (25)	1 (8)	4 (33)	2 (17)		1 (8)
*Moderate*							
*Severe*							

Systemic AE							
Pyrexia							
*Mild*	2 (33)		2 (17)	3 (25)	2 (17)		
*Moderate*		2 (17)				1 (8)	
*Severe*							
Feverishness							
*Mild*	1 (17)	2 (17)	3 (25)	4 (33)	5 (42)	3 (25)	5 (42)
*Moderate*		2 (17)	2 (17)	1 (8)		1 (8)	1 (8)
*Severe*		1 (8)	1 (8)	1 (8)			
Arthralgia							
*Mild*	1 (17)	3 (25)	3 (25)	2 (17)	2 (17)		2 (17)
*Moderate*			2 (17)	1 (8)	1 (8)		1 (8)
*Severe*							
Myalgia							
*Mild*	2 (33)	7 (58)	4 (33)	5 (42)	3 (25)	1 (8)	5 (42)
*Moderate*		1 (8)	3 (25)	2 (17)	2 (17)	1 (8)	1 (8)
*Severe*				1 (8)			
Fatigue							
*Mild*	2 (33)	5 (42)	5 (42)	2 (17)	4 (33)	3 (25)	3 (25)
*Moderate*		1 (8)	2 (17)	5 (42)			1 (8)
*Severe*		1 (8)	1 (8)				
Headache							
*Mild*	1 (17)	7 (58)	3 (25)	3 (25)	4 (33)	2 (17)	4 (33)
*Moderate*		1 (8)	3 (25)	3 (25)	2 (17)	2 (17)	1 (8)
*Severe*		1 (8)		1 (8)			
Nausea							
*Mild*	1 (17)	2 (17)	4 (33)	6 (50)	2 (17)		1 (8)
*Moderate*		2 (17)				1 (8)	2 (17)
*Severe*							
Malaise							
*Mild*	2 (33)	3 (25)	3 (25)	2 (17)	2 (17)	3 (25)	3 (25)
*Moderate*		3 (25)	2 (17)	2 (17)		1 (8)	2 (17)
*Severe*				1 (8)			

Unsolicited AEs							
*Mild*	4 (67)	4 (33)	5 (42)	2 (17)	4 (33)	3 (25)	3 (25)
*Moderate*	1 (17)		1 (8)	1 (8)	1 (8)		2 (17)

Frequency of unsolicited AEs, including abnormalities of blood safety profile, considered to be related to vaccination are included in Table 1. A volunteer in Group A reported transient axillary lymphadenopathy. There were 9 haematological AEs considered to be related to ChAdOx1 85A vaccination (lymphopaenia (3 mild), neutropaenia (1 mild, 1 moderate), leukopaenia (1 mild), eosinophilia (2 mild) and thrombocytopaenia (1 mild)) and 5 related to MVA85A (lymphopaenia (1 mild, 1 moderate), leukopaenia (2 mild) and eosinophilia (1 mild)). One volunteer in Group C had a moderate lymphopaenia considered possibly related to MVA85A which was ongoing at the end of the study. All others resolved fully.

A Group C volunteer reported shingles in the T9 dermatomal distribution at D56, 28 days post D28 vaccination with ChAdOx1 85A. Due to the timing of the event and past medical history (volunteer had experienced two previous episodes of shingles several years prior to enrolment) this was deemed not related to vaccination.

There were no Serious Adverse Events (SAEs) reported during the course of the study in any group.

### 3.2 Vaccine immunogenicity

#### 3.2.1 Vaccination with ChAdOx1 85A boosted *ex-vivo* Ag85A-specific IFN-γ responses that are further boosted by MVA85A

**Fig. 2:**
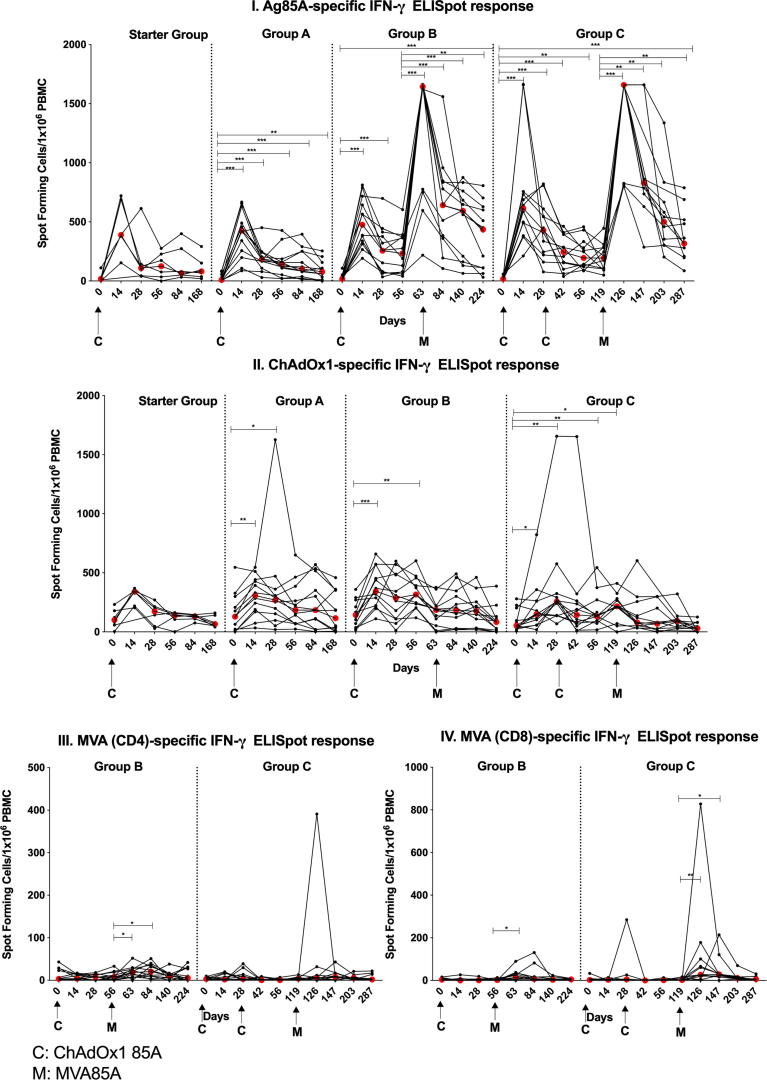
*Ex vivo* PBMC IFN-*γ* ELISpot responses (Spot Forming cells (SFC)/1 × 10^6^ PBMC) to Ag85A pool of 66 peptides (I) and ChAdOx1 (II) in BCG-vaccinated healthy UK adults vaccinated with 5 × 10^9^ vp ChAdOx1 85A (Starter Group (D0)) and 2.5 × 10^10^ vp (Groups A (D0), B (D0) and C (D0 and D28)). Group B and C were vaccinated with MVA85A (1 × 10^8^ pfu) at D56 and D119 respectively. Results for IFN-*γ* responses to MVA peptides for CD4 (III), and CD8 epitopes (IV) are presented for study groups B and C. Individual values are shown for each volunteer. Red dots indicate median values. The Wilcoxon matched pairs signed rank test was used for comparing responses to baseline in each group. Significant differences between groups are as follows: *P ≤ 0.05, **P ≤ 0.01, ***P ≤ 0.001.

A single dose (2.5 × 10^10^ vp) of ChAdOx1 85A induced a significant increase in Ag85A-specific *ex-vivo* ELISpot responses in Groups A, B and C compared to baseline. Responses peaked at D14 (Fig. 2I; p = 0.0005 for Groups A, B and C), and remained significantly higher than baseline until the end of follow-up (D168 in Group A (p = 0.003). A second dose of ChAdOx1 85A (Group C) did not further boost these responses (Fig. 2I), however they were significantly boosted with MVA85A vaccination at D56 and D119 in Group B and Group C volunteers respectively (p = 0.0005 for Group B D63 and p = 0.001 for Group C D126, (Fig. 2I)) remaining significantly above baseline at the end of the study (Group B D224 p = 0.0005; for; Group C D287 p = 0.001). No difference was detected in the magnitude of the MVA85A-boosted responses between Group B and Group C one week post-MVA85A (p = 0.2274).

AUC analysis for Ag85A-specific responses was performed on groups B and C and volunteers from a previous trial, TB022, who received MVA85A alone by intramuscular injection [20]. To account for the different time points in the groups, analysis was only performed using four time points, starting from 1 week post-MVA85A vaccination and including 4, 12 and 24 weeks post-MVA85A vaccination. Groups B and C had a median AUC of 14985 and 14978 SFC/1 × 10^6^ PBMC respectively, compared with 9527 SFC/1 × 10^6^ PBMC in the MVA85A alone group, although the difference between the groups was not significant.

An increase in Ag85A-specific responses was detected in the Starter Group but did not reach statistical significance. AUC analysis comparing the Starter Group with Group A was not significant

Baseline PPD-specific IFN-γ responses were not boosted by ChAdOx1 85A in the Starter Group or groups A and C, however group B had a significant increase in responses between D0 and D14 (p = 0.007) (Supplementary Table 2). Boosting with MVA85A significantly increased PPD-specific IFN-γ responses at D63 in Group B (p = 0.005) and D126 in Group C (p = 0.002), contracting to baseline at D84 and D147 (Groups B and C respectively). MVA85A boosted responses were not significantly different in Group B (D63) and Group C (D126).

ChAdOx1-specific responses were detected in Groups A, B and C (Fig. 2II) peaking at D14 (Group A p = 0.0015; Group B p = 0.0005; Group C p = 0.0278). Group A remained significantly higher until D28 (p = 0.027), Group B until D56 (p = 0.005) and Group C until D119 (p = 0.024). A second dose of ChAdOx1 85A (Group C) did not enhance this anti-vector specific response.

No MVA-specific responses were detectable in Starter Group and Group A. Group B and C had significantly higher responses to the MVA CD8+ T cell epitopes one week following MVA85A vaccination (Group B p = 0.034; Group C p = 0.0098). Responses to CD4+ T cell epitopes were detectable in Group B, one and four weeks post-MVA85A vaccination (p = 0.033 and p = 0.012 respectively) (Fig. 2III).

There was no statistical association between ELISpot insert and vector responses: For ChAdOx1 vs Ag85A at D14 for Groups A, B and C combined and at D42, following a second ChadOx1 85A dose, in Group C and for MVA vs Ag85A at D 63 (Group B) and D126 (Group C), all p > 0.05 (Spearman test).

#### 3.2.2 Vaccination with ChAdOx1 85A induces intracellular cytokine responses in Ag85A-specific CD4+ and CD8+ T cells

**Fig. 3:**
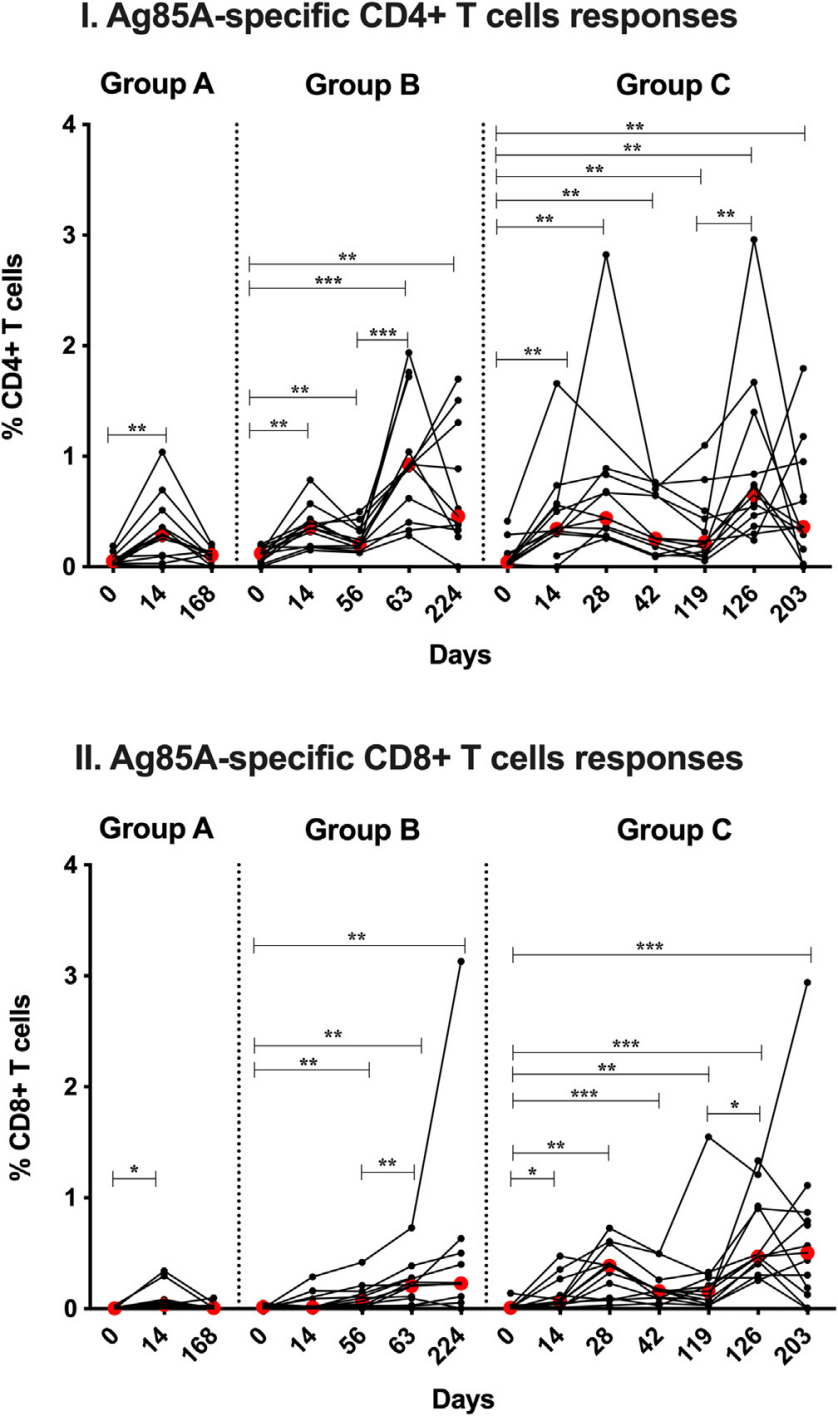
Whole blood ICS Ag85A-specific responses in volunteers vaccinated with ChadOx1 85A (2.5 × 10^10^ vp) (Groups A (D0), B (D0) and C (D0 and D28)). Group B and C were vaccinated with MVA85A (1 × 10^8^ pfu) at D56 and D119 respectively. Percentages of CD4+ T cells producing IFN-*γ*, TNF-*α*, IL-2 and IL-17 (summed responses) are shown in figure (I) and Percentages of CD8+ T cells producing IFN-*γ* and TNF-*α* (summed responses) are shown in figure (II). Individual values are shown for each volunteer. Red dots indicate median values in each group. The Wilcoxon matched pairs signed rank test was used to detect differences between time points in the same group. Significant differences between groups are as follows: *P ≤ 0.05, **P ≤ 0.01, ***P ≤ 0.001.

ChAdOx1 85A induced Ag85A specific IFN-γ+, TNF-α+, IL-2+ and IL-17+ CD4+ T cells in Groups A, B and C at D14 (Fig. 3I; p = 0.002) which remained significantly higher at D56 in Group B (p = 0.002). Boosting with MVA85A further enhanced this response in Groups B and C (D63 p = 0.001; D126 p = 0.003 respectively) and were comparable between the two groups (p = 0.379), remaining significantly high at the end of follow-up (Group B: D0 v D224 p = 0.004; Group C: D0 v D203 p = 0.005).

Ag85A-specific IFN-γ+, TNF-α+ CD8+ T cells were induced by ChAdOx1 85A vaccination (Fig. 3II) and were boosted by MVA85A (Group B: D63 p = 0.008; Group C: D126 p = 0.019). These responses were higher in group C than in group B (p = 0.0007) and remained significantly higher at the end of the follow-up for both groups (Group B: D0 vs D224 p = 0.01; Group C: D0 vs D203 p = 0.001).

#### 3.2.3 Polyfunctional CD4+ and CD8+ T cells are detectable after ChAdOx1 85A and MVA85A vaccinations

**Fig. 4:**
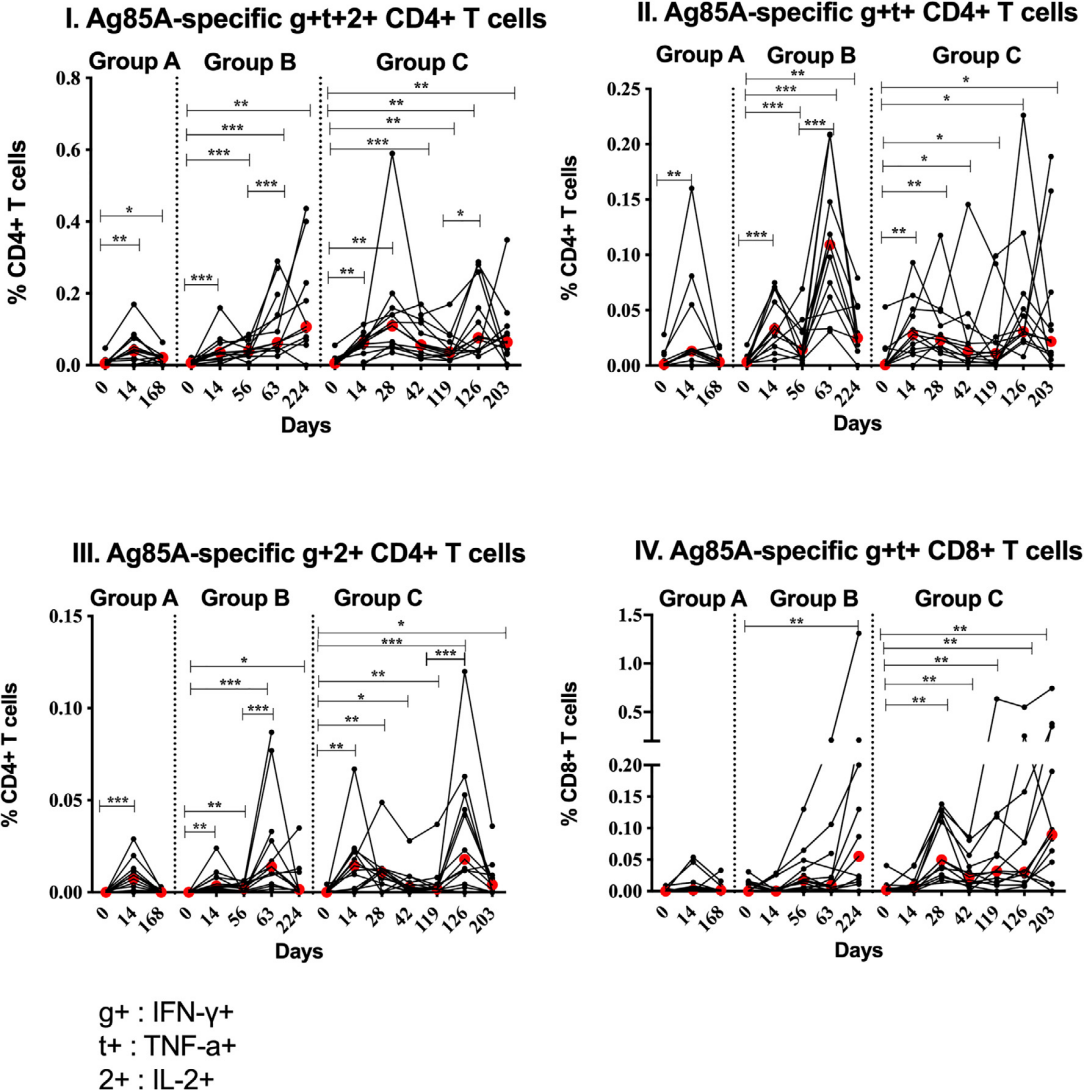
Polyfunctionality of Ag85A-specific CD4+ (I-III) and CD8+ (IV) T cells in volunteers vaccinated with ChadOx1 85A (2.5 × 10^10^ vp) (Groups A (D0), B (D0) and C (D0 and D28)). Group B and C were vaccinated with MVA85A (1 × 10^8^ pfu) at D56 and D119 respectively. Percentages of CD4+ T cells simultaneously producing IFN-*γ* (g+), TNF-*α* (t+) and IL-2 (2+), positive for IFN-*γ* and TNF-*α* and positive for IFN-*γ* and IL-2 are shown in figures (I), (II) and (III) respectively. Percentages of CD8+ T cells producing both IFN-γ and TNF-α are shown in figure (IV). Individual values are shown for each volunteer. Red dots indicate median values in each group. The Wilcoxon matched pairs signed rank test was used to detect differences between time points in the same group. Significant differences between groups are as follows: *P ≤ 0.05, **P ≤ 0.01, ***P ≤ 0.001. Results of the detected cytokines combinations are presented.

Ag85A-specific CD4+ T cells producing combinations of IFN-γ, TNF-α and IL-2 (g+t+2+ ) (Fig. 4I), IFN-γ and TNF-α (g+t+) (Fig. 4II) and IFN-γ and IL2 (g+2+) (Fig. 4III) were induced by ChAdOx1 85A in Groups A, B and C (Group A p = 0.005, Group B p = 0.001 and Group C p = 0.002 for g+t+2+; Group A p = 0.004, Group B p = 0.001 and Group C p = 0.0039 for g+t+; Group A p = 0.001, Group B and C p = 0.002 for g+2+ all D14 compared to D0). Ag85A-specific polyfunctional CD4+ T cell cytokine responses were enhanced further by MVA85A at D63 compared to D56 in Group B (p = 0.001 for g+t+2+, g+t+ and g+2+) and at D126 compared to D119 in Group C (p = 0.021 for g+t+2+ and p = 0.001 for g+2+). A second ChAdOx1 85A vaccination did not boost the frequency of the Ag85A-specific polyfunctional CD4+ T cell responses. Ag85A-specific g+t+CD8+ T cells were significantly higher than D0 at D224 in Group B (p = 0.0098) and at D28, D42, D126 and D203 (p = 0.002) and at D119 (p = 0.005) in Group C (Fig. 4IV). Results of the detected cytokines combinations are presented.

#### 3.2.4 Immunoglobulin G (IgG) responses following ChAdOx1 85A and MVA85A vaccination

**Fig. 5:**
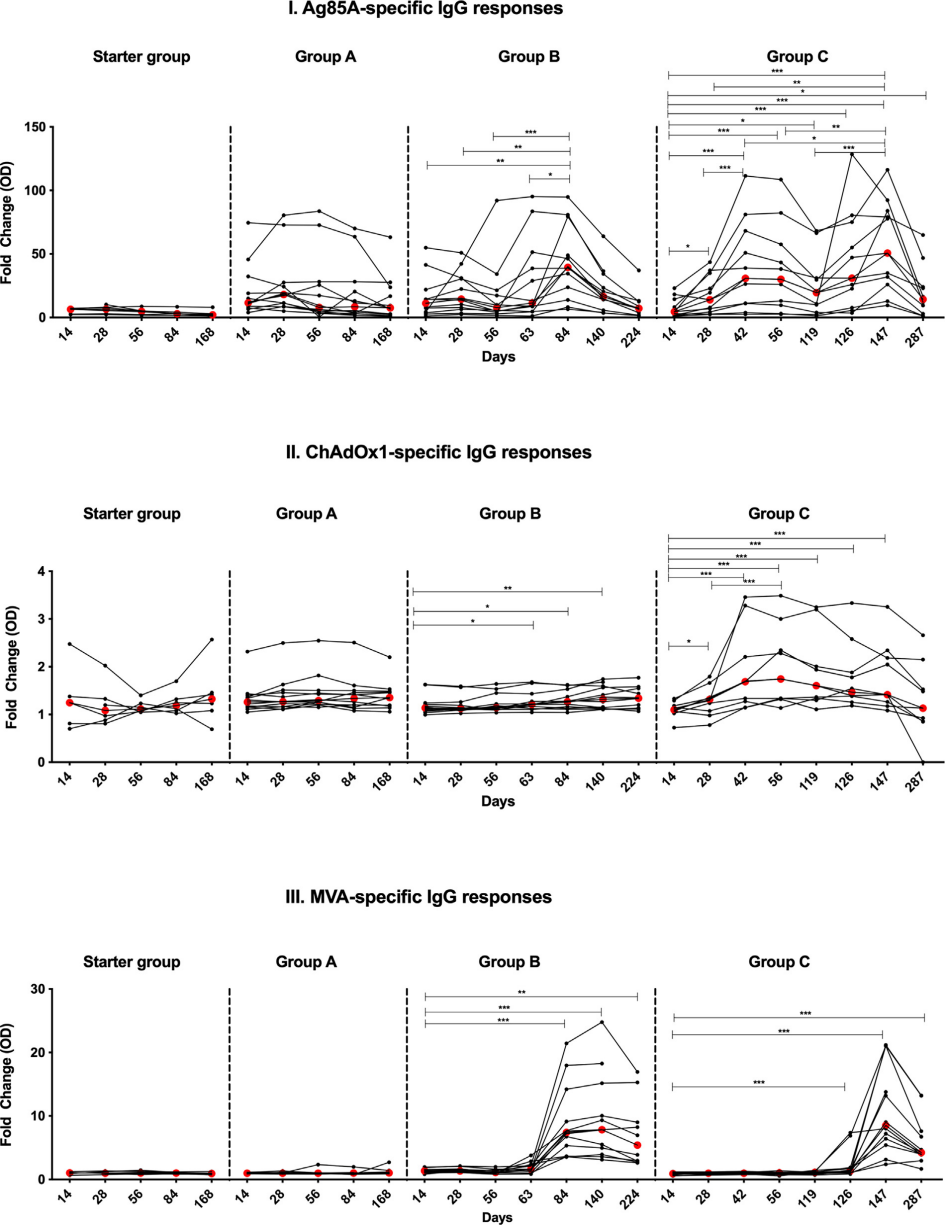
Serum antibody IgG responses in volunteers vaccinated with ChAdOx1 85A (Starter Group with 2.5 × 10^9^ vp at D0), (A, B and C with 5 × 10^10^ vp at D0 for all group in addition to D28 in Group C). Groups A and B volunteers received MVA85A (1 × 10^8^ pfu) at D56 and D119 respectively. Figure (I) shows responses to recombinant Ag85A, Figure (II) shows ChAdOx1-specific IgG responses and figure (III) presents anti-MVA IgG responses. Data is presented as fold change responses calculated by dividing each time point's antibody response (measured in optical density) by its corresponding D0 response. Individual values are shown for each volunteer. Red dots indicate median values in each group. The Wilcoxon matched pairs signed rank test was used to detect differences between time points in the same group. Significant differences between groups are as follows: *P ≤ 0.05, **P ≤ 0.01, ***P ≤ 0.001.

**Supplementary figure 3:**
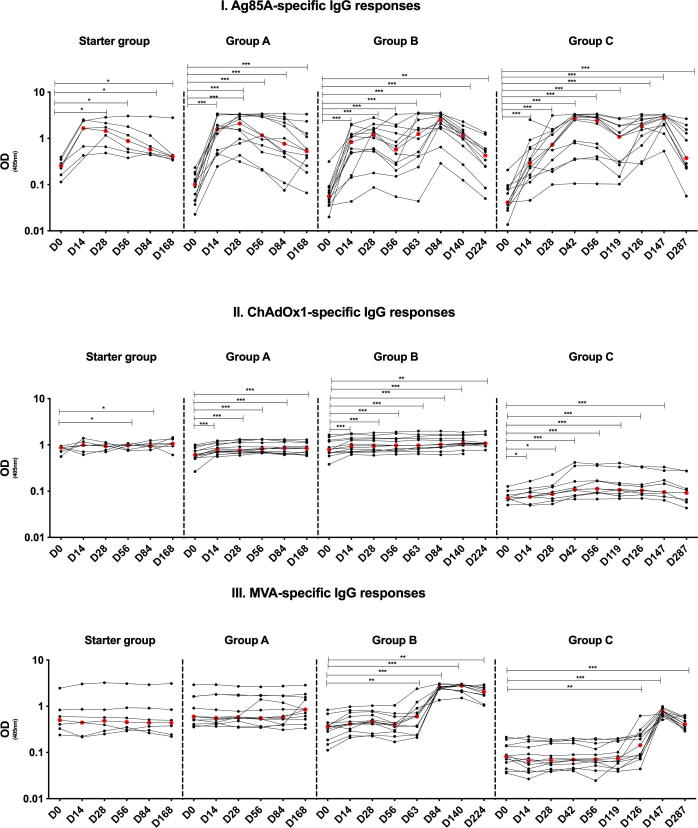
Serum antibody IgG responses in volunteers vaccinated with ChAdOx1 85A (Starter Group with 2.5 × 109 vp at D0), (A, B and C with 5 × 1010 vp at D0 for all group in addition to D28 in Group C). Groups A and B volunteers received MVA85A (1 × 108 pfu) at D56 and D119 respectively. Figure (I) shows responses to recombinant Ag85A, Figure (II) shows ChAdOx1-specific IgG responses and figure (III) presents anti-MVA IgG responses. Data is presented in Optical Density (OD) measured at 405nm. Individual values are shown for each volunteer. Red dots indicate median values in each group. The Wilcoxon matched pairs signed rank test was used to detect differences between time points in the same group. Significant differences between groups are as follows: *P ≤ 0.05, **P ≤ 0.01, ***P ≤ 0.001.

Ag85A-specific IgG responses were induced by 5 × 10^9^ vp ChAdOx1 85A, peaking at D28 and remaining higher than baseline at D168 (Starter Group p = 0.031 for all time points). A similar pattern was observed with 2.5 × 10^10^ vp ChAdOx1 85A, with responses being significantly higher at D14 than D0 (p = 0.0005 for all time points up to D84 and p = 0.001 at D168 (Group A), P = 0.0005 up to D140 and p = 0.002 for D224 (Group B) and p = 0.0005 for all time points in Group C up to D147 and p = 0.001 for D287. The magnitude of the measured IgG response was not ChAdOx1 85A dose-dependent (Supplementary Fig. 3I). Boosting with MVA85A resulted in increased IgG fold change in Group B at D84 (D84 v D14 p = 0.009; D84 v D28 p = 0.005 and D84 v D56 p = 0.0005) and Group C at D147 (D147 v D14 p = 0.001; D147 v D28 p = 0.002; D147 v D42 p = 0.024, D147 v D56 p = 0.0098 and D147 v D119 p = 0.001). Boosting with ChAdOx1 85A at D28 enhanced the IgG fold change in Group C (p = 0.001 at D42) (Fig. 5I). Ag85A-specific IgG responses were not significantly different between Groups B and C (p > 0.05 at 1 and 4 weeks post-MVA85A vaccination) (Supplementary Fig. 3I and Fig. 5I).

Vaccination with 5 × 10^9^ vp ChAdOx1 85A induced significant ChAdOx1-specific IgG in the Starter Group (p = 0.031 at D56 and D84). ChAdOx1 85A at a dose of 2.5 × 10^10^ vp induced significant ChAdOx1-specific IgG in Groups A, B and C (Group A p = 0.0005 at D14 up to D84 and p = 0.001 at D168; Group B p = 0.001 at D14, p = 0.0005 at D28 up to D140 and p = 0.002 at D224; Group C p = 0.02 at D14, p = 0.012 at D28, p = 0.0005 at D42 up to D147) (Supplementary Fig. 3II). Vaccine-induced anti-ChAdOx1 IgG fold change was significant in Groups B and C. The induced ChAdOx1 response was significantly higher at D63 and remained high up to D140 in Group B (D63 p = 0.021; D84 p = 0.027 and D140 p = 0.007 all compared to D14). Significantly higher fold change IgG response was observed at all time points up to D147 compared to D14 in Group C (D28 p = 0.016, D56 p = 0.001 and p = 0.0005 for all other time points up to D147). Two doses of ChAdOx1 85A induced higher fold change in Group C (D28 vs D56 peak fold change p = 0.0005; Fig. 5II).

MVA85A vaccination induced anti-MVA IgG that peaked two weeks after vaccination and remained significantly higher than D0 in both Groups B (D63 p = 0.005; D84 and D140 p = 0.0005 and D224 p = 0.002) and Group C (D126 p = 0.002, D147 p = 0.0005 and D287 p = 0.001) (Supplementary Fig. 3III). Fold change MVA-specific IgG response was significantly induced in both Groups B and C (p = 0.0005 for D84 and D140 Group B and D147 Group C; p = 0.002 for D224 Group B and p = 0.001 for D126 and D287 Group C) (Fig. 5III).

## 4 Discussion:

In this phase I first-in-human clinical trial, we found that vaccination with candidate TB vaccine ChAdOx1 85A was well tolerated up to a dose of 2.5 × 10^10^ vp, with an acceptable safety profile across all groups.

Vaccination with ChAdOx1 85A induced Ag85A-specific IFN-γ responses. As expected from previous studies, these responses were boosted with MVA85A vaccination, and the direct effect of homologous boosting with ChAdOx1 85A was minimal. AUC analysis of the Ag85A-specific IFN-γ ELISpot response 1-24 weeks after vaccination with MVA85A showed no significant difference between Group B and Group C, although both had higher median AUC responses than a previous trial of MVA85A when given alone. We have previously shown that potent Ag85A-specific immune responses were induced by MVA85A boosting of AERAS-402 vaccine in healthy UK adults, the magnitude of these boosted responses in the group that received two doses of AERAS-402 was comparable to those detected in Group C of the present trial (p = 0.176) and to a single dose of MVA85A (p = 0.728) [14], [20]. The critical role of IFN-γ in control of TB has been reported in humans and animal models and we have shown that vaccine-induced *Mycobacteria*-specific IFN-γ secreting cells are associated with reduced risk of TB disease [21], [22], [23], [24]. ChAdOx1 85A did not enhance PPD IFN-γ responses; this might be attributed to the high PPD responses detected at baseline, which are expected in BCG-vaccinated individuals. Other cytokines are shown to play a role in protection against TB; blocking TNF-α is reported to be associated with susceptibility to TB [25], IL-2, another Th1 cytokine, induces proliferation and maintenance of effector CD4+ and CD8+ T cells [26] and IL-17 has been associated with protection against TB in mice and humans [27], [28]. Production of these cytokines was also induced by ChAdOx1 85A and boosted by MVA85A. Studies in humans and animals have suggested a significant role of CD8+ T cells in control of TB [29], [30], [31]. Vaccination with ChAdOx1 85A resulted in significant Ag85A-specific IFN-γ+ TNF-α+ CD8+ T cells which were boosted by MVA85A, previously reported to be a poor inducer of CD8+ T cells responses [20]. The responses in subjects boosted with MVA85A were higher than those in the ChAdOx1 85A alone group, reflecting the superior immunogenicity of a prime-boost combination reported for other pathogens [13], [16], [17], [32], [33], [34].

Polyfunctional T cells have been shown to play a role in protection against intracellular pathogens and were shown to be induced by MVA85A vaccination [35], [36], [37]. Here we demonstrate that polyfunctional CD4+ T cells are induced by ChAdOx1 85A vaccination and homologous boosting was required to obtain polyfunctional CD8+ T cell responses, but had minimal effect on the detected polyfunctional CD4+ T cells responses.

Immune responses to viral vectors may potentially modify Ag85A-specific responses [38], to this end we have tested vector-specific cellular and humoral responses. Low baseline (D0) vector-specific IFN-γ responses were detected in volunteers from the 4 study groups (median ELISpot responses of 3 SFC/1 × 10^6^ PBMC for MVA CD4, 4 SFC/1 × 10^6^ PBMC for MVA CD8 of and 102 SFC/1 × 10^6^ PBMC for ChAdOx1). A single dose of ChAdOx1 85A induced ChAdOx1-specific IFN-γ responses but were not boosted by homologous vaccination. MVA-specific IFN-γ responses were detected following MVA85A vaccination as we have shown previously [14]. The detected cellular anti-vector responses were not associated with the vaccine-induced insert-specific responses.

Although the role of antibodies in control of TB is unclear, some studies have demonstrated a crucial role of antibodies in protection against infection and we have demonstrated that vaccine-induced Ag85A-specific IgG is associated with a reduced risk of TB disease [24], [39]. Here, we show that vaccination with ChAdOx1 85A enhances Ag85A-specific IgG responses, which in turn are boosted by MVA85A. IgG antibodies to the vectors were detected following vaccination; however no association between anti-vector and anti-Ag85A IgG responses is evident.

Whilst it is likely that more than one antigen is needed in a subunit vaccine for protection in genetically diverse human populations, this experimental medicine study demonstrates proof-of-concept of a prime-boost approach with a novel simian adenoviral vector prime and an MVA boost in BCG-vaccinated adults. Further work is needed to identify novel antigenic targets for inclusion in a multi-antigenic prime-boost vaccination regimen.

## 5 Conclusion

This is the first study of the safety and immunogenicity of ChAdOx1 85A in humans. We have shown the vaccine to be well-tolerated when given alone or in combination with MVA85A, and that ChAdOx1 85A induces potent Ag85A specific CD4+ and CD8+ T cell responses which are boosted by MVA85A vaccination.

## Financial support statement

This research was supported by the Wellcome Trust (grant 095780/Z/11/Z awarded to HMcS), the National Institute for Health Research (NIHR) Oxford Biomedical Research Centre based at Oxford University Hospitals NHS Trust and University of Oxford, the European Union’s Horizon 2020 research and innovation programme under grant agreement No 643381 (TBVAC2020), Aeras (MCA002), and the National Institute for Health Research Clinical Research Network Thames Valley and South Midlands (TVCLRN), and carried out at the University of Oxford and the National Institute for Health Research (NIHR)/Wellcome Trust Birmingham Clinical Research Facility. The views expressed are those of the authors and not necessarily those of the NHS, the NIHR or the Department of Health. HMcS is a Jenner Institute Investigator.

## Declaration of Competing Interest

The authors declare that they have no known competing financial interests or personal relationships that could have appeared to influence the work reported in this paper.

## References

[1] WHO, Global tuberculosis report 2016; 2017.

[2] The Global Plan to End TB: The Paradigm Shift 2016–2020. Stop TB Partnership; 2016.

[3] Colditz G.A. (1994). Efficacy of BCG vaccine in the prevention of tuberculosis: meta-analysis of the published literature. JAMA.

[4] Trunz B.B., Fine P., Dye C. (2006). Effect of BCG vaccination on childhood tuberculous meningitis and miliary tuberculosis worldwide: a meta-analysis and assessment of cost-effectiveness. The Lancet.

[5] Rodrigues L.C., Diwan V.K., Wheeler J.G. (1993). Protective effect of BCG against tuberculous meningitis and miliary tuberculosis: a meta-analysis. Int J Epidemiol.

[6] McShane H. (2005). Boosting BCG with MVA85A: the first candidate subunit vaccine for tuberculosis in clinical trials. Tuberculosis.

[7] Xing Z., Charters T.J. (2007). Heterologous boost vaccines for bacillus Calmette-Guérin prime immunization against tuberculosis. Expert Rev Vaccines.

[8] Vordermeier H.M. (2004). Cellular immune responses induced in cattle by heterologous prime–boost vaccination using recombinant viruses and bacille Calmette-Guérin. Immunology.

[9] Vordermeier H.M. (2006). Immune responses induced in cattle by vaccination with a recombinant adenovirus expressing mycobacterial antigen 85A and mycobacterium bovis BCG. Infect Immun.

[10] McShane H. (2001). Enhanced immunogenicity of CD4+ T-Cell responses and protective efficacy of a DNA-modified vaccinia virus ankara prime-boost vaccination regimen for murine tuberculosis. Infect Immun.

[11] Tameris M.D. (2013). Safety and efficacy of MVA85A, a new tuberculosis vaccine, in infants previously vaccinated with BCG: a randomised, placebo-controlled phase 2b trial. The Lancet.

[12] Ogwang C. (2015). Prime-boost vaccination with chimpanzee adenovirus and modified vaccinia Ankara encoding TRAP provides partial protection against Plasmodium falciparum infection in Kenyan adults. Sci Transl Med.

[13] Ewer K.J. (2013). Protective CD8+ T-cell immunity to human malaria induced by chimpanzee adenovirus-MVA immunisation.

[14] Sheehan S. (2015). A phase I, open-label trial, evaluating the safety and immunogenicity of candidate tuberculosis vaccines AERAS-402 and MVA85A, administered by prime-boost regime in BCG-vaccinated healthy adults. PLoS ONE.

[15] Van Der Meeren O. (2018). Phase 2b controlled trial of M72/AS01E vaccine to prevent tuberculosis. N Engl J Med.

[16] Dicks M.D. (2012). A novel chimpanzee adenovirus vector with low human seroprevalence: improved systems for vector derivation and comparative immunogenicity. PLoS ONE.

[17] Antrobus R.D. (2014). Clinical assessment of a novel recombinant simian adenovirus ChAdOx1 as a vectored vaccine expressing conserved Influenza A antigens. Mol Ther.

[18] Stylianou E. (2015). Improvement of BCG protective efficacy with a novel chimpanzee adenovirus and a modified vaccinia Ankara virus both expressing Ag85A. Vaccine.

[19] Hanekom W.A. (2004). Novel application of a whole blood intracellular cytokine detection assay to quantitate specific T-cell frequency in field studies. J Immunol Methods.

[20] Meyer J. (2013). Comparing the safety and immunogenicity of a candidate TB vaccine MVA85A administered by intramuscular and intradermal delivery. Vaccine.

[21] Jouanguy E. (1996). Interferon-gamma-receptor deficiency in an infant with fatal bacille Calmette-Guerin infection. N Engl J Med.

[22] Newport M.J. (1996). A mutation in the interferon-gamma-receptor gene and susceptibility to mycobacterial infection. N Engl J Med.

[23] Flynn J.L. (1993). An essential role for interferon gamma in resistance to Mycobacterium tuberculosis infection. J Exp Med.

[24] Fletcher H.A. (2016). T-cell activation is an immune correlate of risk in BCG vaccinated infants. Nat Commun.

[25] Harris J., Keane J. (2010). How tumour necrosis factor blockers interfere with tuberculosis immunity. Clin Exp Immunol.

[26] Smith K.A. (1988). Interleukin-2: inception, impact, and implications. Science.

[27] Khader S.A. (2007). IL-23 and IL-17 in the establishment of protective pulmonary CD4+ T cell responses after vaccination and during Mycobacterium tuberculosis challenge. Nat Immunol.

[28] Babu S. (2010). Regulatory T cells modulate Th17 responses in patients with positive tuberculin skin test results. J Infect Dis.

[29] Lewinsohn D.A. (2003). Mycobacterium tuberculosis-specific CD8+ T cells preferentially recognize heavily infected cells. Am J Respir Crit Care Med.

[30] Chen C.Y. (2009). A critical role for CD8 T cells in a nonhuman primate model of tuberculosis. PLoS Pathog.

[31] Sousa A.O. (2000). Relative contributions of distinct MHC class I-dependent cell populations in protection to tuberculosis infection in mice. Proc Natl Acad Sci USA.

[32] Milligan I.D. (2016). Safety and immunogenicity of novel adenovirus type 26– and modified vaccinia ankara–vectored ebola vaccines: a randomized clinical trial. JAMA.

[33] Sheehy S.H. (2012). Phase Ia clinical evaluation of the safety and immunogenicity of the Plasmodium falciparum blood-stage antigen AMA1 in ChAd63 and MVA vaccine vectors. PLoS ONE.

[34] Ewer K. (2016). A monovalent chimpanzee adenovirus Ebola vaccine boosted with MVA. N Engl J Med.

[35] Beveridge N.E. (2007). Immunisation with BCG and recombinant MVA85A induces long-lasting, polyfunctional Mycobacterium tuberculosis-specific CD4+ memory T lymphocyte populations. Eur J Immunol.

[36] Lindenstrom T. (2009). Tuberculosis subunit vaccination provides long-term protective immunity characterized by multifunctional CD4 memory T cells. J Immunol.

[37] Darrah P.A. (2007). Multifunctional TH1 cells define a correlate of vaccine-mediated protection against Leishmania major. Nat Med.

[38] Saxena M. (2013). Pre-existing immunity against vaccine vectors–friend or foe?. Microbiology.

[39] Jacobs A.J. (2016). Antibodies and tuberculosis. Tuberculosis (Edinb).

