# Supplementary material for: A phase I trial evaluating the safety and immunogenicity of a candidate tuberculosis vaccination regimen, ChAdOx1 85A prime – MVA85A boost in healthy UK adults
Source: Vaccine. 2020 Jan 22;38(4):779–89. doi: 10.1016/j.vaccine.2019.10.102 (PMC6985898; doi:10.1016/j.vaccine.2019.10.102)
Supplement: Supplementary data 1 [file mmc1.docx]

**Supplementary Table 1:** Volunteer baseline demographics by group. Age and years since BCG compared across groups using one-way ANOVA, Tukey’s multiple comparisons test. There was no significant difference between groups (p > 0.05).

|  | Starter Group (*n*=6) | Group A (*n*=12) | Group B (*n*=12) | Group C (*n*=12) |
| --- | --- | --- | --- | --- |
| Age  *Mean age, years (range)* | 33 (22-54) | 27 (20-47) | 30 (20-51) | 30 (20-47) |
| Sex  *Female, n (%)* | 6 (100) | 6 (50) | 8 (67) | 6 (50) |
| Country of birth  *UK:Other* | 4:2 | 12:0 | 8:4 | 11:1 |
| Years since BCG  *Mean time, years (range)* | 18 (12-25) | 13 (1-32) | 23 (4-42) | 19 (9-34) |

**Supplementary Table 2**: *Ex-vivo* ELISpot responses (Spot Forming cells (SFC)/1x10^6^ PBMC) to Purified Protein Derivative (PPD): Minimum, maximum, medians, 25^th^ and 75^th^ percentiles are presented

| Group | Starter: ChAdOx1 85A (5x10^9^ vp) | | | | | |  |  |  |  |
| --- | --- | --- | --- | --- | --- | --- | --- | --- | --- | --- |
| Days | 0 | 14 | 28 | 56 | 84 | 168 |  |  |  |  |
| Minimum | 13 | 86 | 16 | 13 | 46 | 34 |  |  |  |  |
| 25% Percentile | 182.5 | 141.5 | 20.5 | 167.5 | 85.75 | 118 |  |  |  |  |
| Median | 385.5 | 312 | 205 | 289 | 423.5 | 361 |  |  |  |  |
| 75% Percentile | 595.75 | 533.5 | 600.5 | 409 | 509 | 524.75 |  |  |  |  |
| Maximum | 604 | 684 | 626 | 532 | 578 | 713 |  |  |  |  |
|  |  |  |  |  |  |  |  |  |  |  |
| Group | A: ChAdOx1 85A (2.5x10^10^ vp) | | | | | |  |  |  |  |
| Days | 0 | 14 | 28 | 56 | 84 | 168 |  |  |  |  |
| Minimum | 13 | 24 | 11 | 8 | 18 | 0 |  |  |  |  |
| 25% Percentile | 26.25 | 71 | 73.75 | 29 | 30 | 21 |  |  |  |  |
| Median | 69.5 | 174.5 | 178 | 90 | 99.5 | 118.5 |  |  |  |  |
| 75% Percentile | 370.25 | 306.5 | 222.25 | 270 | 379.25 | 362.25 |  |  |  |  |
| Maximum | 730 | 557 | 692 | 654 | 672 | 504 |  |  |  |  |
|  |  |  |  |  |  |  |  |  |  |  |
| Group | B: ChAdOx1 85A (2.5x10^10^ vp) - MVA85A (1x10^8^ pfu) | | | | | | | |  |  |
| Days | 0 | 14 | 28 | 56 | 63 | 84 | 140 | 224 |  |  |
| Minimum | 58 | 109 | 2 | 32 | 94 | 60 | 20 | 21 |  |  |
| 25% Percentile | 109.25 | 158.75 | 46.75 | 131.75 | 301.75 | 118.25 | 90 | 83.25 |  |  |
| Median | 160 | 213.5 | 141.5 | 197 | 542 | 200 | 157.5 | 146 |  |  |
| 75% Percentile | 254.25 | 303.75 | 220.25 | 368 | 690.25 | 423.5 | 362.75 | 251.5 |  |  |
| Maximum | 351 | 630 | 398 | 656 | 923 | 858 | 507 | 510 |  |  |
|  |  |  |  |  |  |  |  |  |  |  |
| Group | C: ChAdOx1 85A (2.5x10^10^ vp) – ChAdOx1 85A (2.5x10^10^ vp) - MVA85A (1x10^8^ pfu) | | | | | | | | | |
| Days | 0 | 14 | 28 | 42 | 56 | 119 | 126 | 147 | 203 | 287 |
| Minimum | 63 | 117 | 23 | 22 | 61 | 0 | 0 | 0 | 0 | 44 |
| 25% Percentile | 104.75 | 174.25 | 113.5 | 55.75 | 99.5 | 109 | 388.75 | 113.5 | 78 | 74 |
| Median | 223.5 | 231 | 282.5 | 112 | 119 | 203 | 667.5 | 275.5 | 191 | 149 |
| 75% Percentile | 366.75 | 438 | 421.5 | 229.25 | 180.75 | 331 | 823.75 | 393.75 | 411 | 238 |
| Maximum | 471 | 537 | 822 | 382 | 356 | 484 | 830 | 568 | 474 | 492 |

**Supplementary Methods 1**

Inclusion Criteria:

Subjects must meet all of the following criteria to enter the trial:

1. Healthy adult aged 18-55 years
2. Resident in or near Oxford (for CCVTM) or Birmingham (for NIHR-WTCRF) and able to travel to Oxford for vaccinations for the duration of the trial period
3. No relevant findings in medical history or on physical examination
4. Confirmation of prior vaccination with BCG not less than 6 months prior to projected trial vaccination date (by visible BCG scar on examination or written documentation)
5. Allow the Investigators to discuss the individual’s medical history with their GP
6. Use effective contraception for the duration of the trial period (females only)
7. Refrain from blood donation during the trial
8. Give written informed consent
9. Allow the Investigator to register subject details with a confidential database to prevent concurrent entry into clinical trials
10. Able and willing (in the Investigator’s opinion) to comply with all the trial requirements

Exclusion Criteria:

Subjects must meet none of the following criteria to enter the trial:

1. Laboratory evidence at screening of latent *M.tb* infection as indicated by a positive IGRA response.
2. Clinical, radiological, or laboratory evidence of current active TB disease
3. Shared a residence within one year prior to day 0 with an individual on anti-tuberculosis treatment or with culture- or smear-positive pulmonary tuberculosis
4. Previous vaccination with any recombinant adenoviral vector (Starter group)
5. Previous vaccination with any recombinant MVA, FP or adenoviral vector (Groups A, B and C)
6. Clinically significant history of skin disorder, allergy, immunodeficiency (including HIV), cancer (except BCC or CIS), cardiovascular disease, gastrointestinal disease, liver disease, renal disease, endocrine disorder, neurological illness, psychiatric disorder, drug or alcohol abuse
7. History of serious psychiatric condition
8. Concurrent oral or systemic steroid medication or the concurrent use of other immunosuppressive agents
9. History of anaphylaxis to vaccination or any allergy likely to be exacerbated by any component of the trial vaccine including eggs
10. Any abnormality of screening blood or urine tests that is deemed to be clinically significant or that may compromise the safety of the subject in the trial
11. Positive HBsAg, HCV or HIV antibodies
12. Female currently lactating, confirmed pregnancy or intention to become pregnant during trial period
13. Use of an investigational medicinal product or non-registered drug, live vaccine, or medical device for 30 days prior to dosing with the trial vaccine, or planned use during the trial period
14. Administration of immunoglobulins and/or any blood products within the three months preceding the planned trial vaccination date
15. Any other significant disease, disorder, or finding, which, in the opinion of the Investigator, may either put the subject at risk or may influence the result of the trial or may affect the subject’s ability to participate in the trial

^a^ Subjects who are excluded from the trial because they have been discovered during screening procedures to be suffering from a previously undiagnosed condition thought to require further medical attention will be referred appropriately to their GP or an NHS specialist service for further investigation and treatment.

^b^ Subjects discovered to have evidence of latent *M.tb* infection as defined by a positive IGRA will be referred for a plain chest x-ray and reviewed with the TB nurse specialists and considered for chemoprophylaxis. If there is any evidence of active TB disease either on clinical or radiological grounds, further investigation and treatment will be offered under the supervision of a consultant physician in respiratory or infectious diseases.
